# ‘We wear too many caps’: role conflict among ambulance service managers

**DOI:** 10.29045/14784726.2019.03.3.4.44

**Published:** 2019-03-01

**Authors:** Joshua Miller

**Affiliations:** West Midlands Ambulance Service University NHS Foundation Trust

## Abstract

**Aims:**

A qualitative study explored how UK ambulance service managers try to identify staff at risk of becoming traumatised by their work, including how they see their role in this task. As research on managers in this field is largely limited to settings outside the NHS, the study was planned as exploratory in nature and developed themes arising from the data.

**Methods:**

Face-to-face, semi-structured interviews were audio-recorded with a purposive sample of six paramedic managers working for an NHS ambulance service. The interview guide included specific questioning on role and identity. The author transcribed these interviews and analysed them using framework analysis. Ethical approval and informed consent were obtained.

**Results:**

The participants were all clinically-trained managers with responsibility for first-line management of front line ambulance crews. They discussed their varying roles both implicitly and explicitly. The roles included: manager, clinician, peer, referrer, ‘adjudicator’, parent figure, ‘the appropriate person’ and the challenger. They discussed the tensions of managing performance and providing emotional support to the same staff, including how some managers perceived this as making staff reluctant to disclose distress. Several participants acknowledged that they were actively creating narratives from different role perspectives, and that readers of the study would also judge them against these different roles.

**Conclusion:**

This study suggests that ambulance service managers within an NHS trust may feel conflicted between varying roles, some relating to their professional identities, and some to work tasks such as performance management and staff support, which may be in tension. Some respondents felt this could make potentially traumatised staff reluctant to disclose distress, which has negative implications in a sector where stress and psychological illness is ascribed as contributing to around 15% of staff sickness. Further research could be conducted into whether this possible role conflict is seen by front line staff as a barrier to disclosing distress.

